# Production of Genistein in *Amaranthus tricolor* var. *tristis* and *Spinacia oleracea* by Expression of *Glycine max Isoflavone Synthase*

**DOI:** 10.3390/plants10112311

**Published:** 2021-10-27

**Authors:** Ashwini Malla, Balamurugan Shanmugaraj, Ashutosh Sharma, Sathishkumar Ramalingam

**Affiliations:** 1 Plant Genetic Engineering Laboratory, Department of Biotechnology, Bharathiar University, Coimbatore 641 046, India; malla164_ashwini@yahoo.co.in (A.M.); balagene3030@gmail.com (B.S.); 2 Tecnologico de Monterrey, School of Engineering and Sciences, Centre of Bioengineering, Campus Queretaro, Av. Epigmenio González No. 500, Fracc. San Pablo, Queretaro 76130, Mexico

**Keywords:** agroinfiltration, *Amaranthus tricolor* var. *tristis*, genistein, isoflavone synthase, metabolic engineering, *Spinacia oleracea*, transient expression

## Abstract

Isoflavonoids, the diverse group of secondary metabolites derived from the phenylpropanoid pathway, are distributed predominantly in leguminous plants. It has received considerable attention in recent days due to its health promoting benefits and is known to prevent certain diseases in humans. These isoflavonoids are synthesized from flavonoid intermediates of phenylpropanoid pathway by the enzyme isoflavone synthase. Metabolic engineering of isoflavonoid biosynthesis in non-legume crop plants could offer the health benefits of these compounds in diverse plant species further contributing for crop improvement. The transient expression of heterologous genes in the host is considered as an alternative to stable expression, that can provide a rapid way of studying the pathway engineering for metabolite production and could also act as a production platform for nutraceuticals and biopharmaceuticals. In this study, isoflavone genistein was produced in *Amaranthus tricolor* var. *tristis* and *Spinacia oleracea* by transiently expressing *Glycine max isoflavone synthase* (*GmIFS*). The *GmIFS* gene was cloned in plant expression vector pEarleyGate 102 HA and pEAQ-HT-DEST 3 and transformed into plants by agroinfiltration. The presence of transgene in the agroinfiltrated leaves was confirmed by semiquantitative reverse-transcription polymerase chain reaction. The flavonoid substrate naringenin and isoflavonoid genistein were quantified using high performance liquid chromatography in both wild-type and infiltrated leaf samples of both the plants. The naringenin content varied in the range of 65.5–338.5 nM/g fresh weight, while the accumulation of genistein was observed with varying concentrations from 113 to 182.6 nM/g fresh weight in the agroinfiltrated leaf samples of both *A. tricolor* var. *tristis* and *S. oleracea*. These results indicate that the transient expression of *GmIFS* gene has led to the synthesis of isoflavonoid genistein in *A. tricolor* var. *tristis* and *S. oleracea* providing an insight that stable expression of this gene could enrich the nutraceutical content in the crop plants. To the best of our knowledge, this is the first report on transient expression of *GmIFS* gene for the production of genistein in *A. tricolor* var. *tristis* and *S. oleracea*.

## 1. Introduction

**Figure 1:**
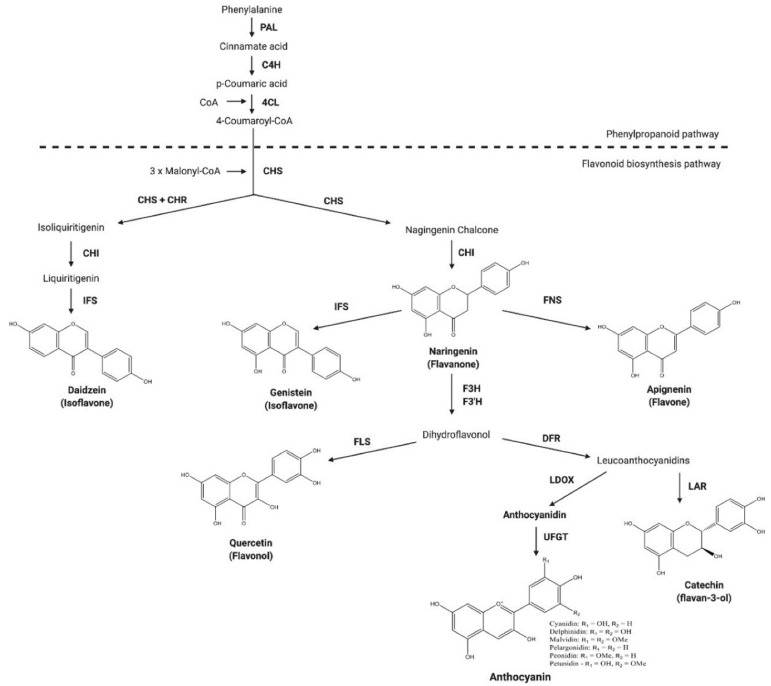
Generalized flavonoid biosynthetic pathway showing the production of genistein from naringenin intermediate. PAL: phenylalanine ammonia-lyase; C4H: cinnamate 4-hydroxylase; 4CL: 4-coumarate-CoA ligase; CHS: chalcone synthase; CHR: chalcone reductase; CHI: chalcone isomerase; IFS: isoflavone synthase; FNS: flavone synthase; FLS: flavonol synthase; F3H: flavanone 3-hydroxylase; F3′H: flavanone 3′-hydroxylase; DFR: dihydroflavonol 4-reductase; LDOX: leucoanthocyanidin dioxygenase; UFGT: UDP-glucose: flavonoid-3-O-glycosyltransferase; and LAR: leucoanthocyanidin reductase (adapted from Ku et al., 2020) [47].

Isoflavonoids, a class of natural plant polyphenols attribute to many biological activities such as antioxidant [1], antimutagenic [2], anticarcinogenic [3,4,5], antiproliferative [6,7] effects are playing an imperative role in human health and nutrition [8,9,10]. These compounds are naturally present in Papilionoideae, a subfamily of Leguminosae or Fabaceae members [11]. Apart from the other vital bioactivities, it is evident that the isoflavonoid genistein in dietary consumption showed a wide spectrum of health protective abilities such as reducing the risk of cardiovascular problems [12], chronic diseases [13], diabetes [14,15,16], cancer [3], osteoporosis [12,14,15,16], obesity [17] and relief from menopausal symptoms [9]. The key enzyme involved in the biosynthesis of isoflavonoids from flavonoid substrates, in the branch of phenylpropanoid pathway, is isoflavone synthase (IFS) (Figure 1), which is natively found in leguminous plants [8,18,19,20,21].

The production of high-value recombinant proteins or nutraceuticals in plants either involves the introduction of new gene into the host species or the overexpression of existing ones in the endogenous pathways. The expression methods used for producing target proteins and metabolites in plants can be either stable or transient expression. Predominantly, the gene manipulation in plants has depended on developing stable transgenic lines for heterologous gene expression. However, it involves tedious laborious and time consuming procedures with requirement of enormous resources. The routine and well-established *Agrobacterium tumefaciens*-mediated gene transfer in plants involves the incorporation of a desired segment of DNA in the binary vector systems and transferred into the plant nuclear genome [22,23]. The recent advances in the transient expression system in plants has led to the remarkable clinches in the scope for producing recombinant proteins, secondary metabolites and other bioactive compounds within a short time in a cost-effective manner [24,25,26,27]. The transient approach of gene expression offers a unique potentiality in the production of foreign proteins at rapid speed with intrinsic scalability [28,29]. The transient expression by agroinfiltration is a mostly preferred method which can be performed either by syringe infiltration or vacuum infiltration [30]. The genus *Nicotiana* is commonly used as an efficient host for transient expression of recombinant proteins due to its higher growth rate, biomass, larger leaf surface area and intrinsic cell architecture that supports the protein processing and compartmentalization [27,31,32]. Further, the transient system has also been used to study gene function and gene silencing, as the process is relatively simple and easy to perform [33].

Green leafy vegetables are regarded as ‘natures antiaging wonders’ due to its medicinal properties [34], nutraceutical values [35] and antioxidant activities [36,37,38] that form the basic needs of mankind and a prerequisite for healthy life. Green leafy vegetables are the major components in traditional diet due to their health benefits, which are mainly due to the rich source of vitamins, fiber, minerals, micronutrients, macronutrients and various phytochemicals [39] with potential antioxidant properties [40,41]. *Amaranthus tricolor* var. *tristis* [42,43] and *Spinacia oleracea* [44] from Amaranthaceae family are used in this study due to the repositories of bioactive compounds with medicinal value, which are known to prevent human diseases, exerting natural biological properties [34].

In this study, an attempt was made to use the green leafy vegetables such as *A. tricolor* var. *tristis* and *S. oleracea* to demonstrate the production of genistein by transient expression of *GmIFS*. Toward this, the *GmIFS* gene was cloned into plant expression vector pEarleyGate 102 HA [45] and pEAQ-HT-DEST 3 [46] and transformed into the plants by agroinfiltration. The infiltrated leaves were harvested, and the metabolites were quantified. The results showed that the transient expression of *GmIFS* can produce isoflavone genistein from the naringenin intermediate in these leafy vegetables.

## 2. Results

**Figure 2:**
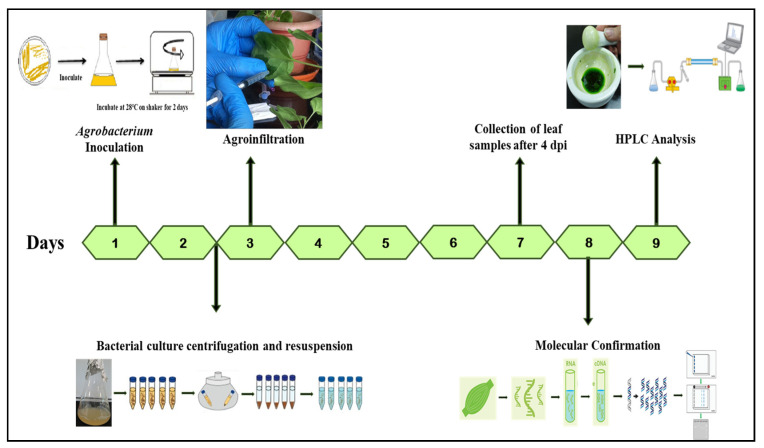
Timeline and diagrammatic representation showing the production of secondary metabolites in plants by transient gene expression.

The seeds of *A. tricolor* var. *tristis* and *S. oleracea* procured from Tamil Nadu Agricultural University seed center were sown in commercially available soil manure mixture for germination and kept in greenhouse. The germinated 15-day-old plantlets were transferred individually to the pots and used for further studies. The schematic representation and timeline for the production of secondary metabolites in plants by transient gene expression are depicted in Figure 2.

### 2.1. Confirmation of Cloned IFS in Plant Expression Vector

**Figure 3:**
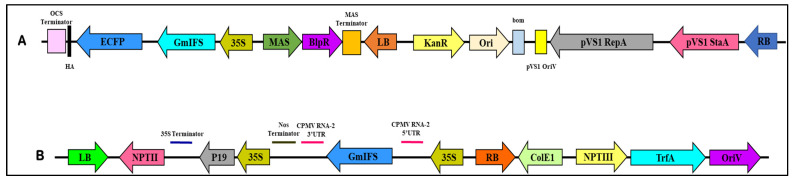
Diagrammatic representation of the gateway destination vectors (**A**) pEarleyGate102 HA and (**B**) pEAQ-HT-DEST 3 used in the study. (**A**) LB and RB: The right and left borders of the T-DNA region in *Agrobacterium*; MAS terminator: mannopine synthase terminator sequence; BlpR: confers resistance to bialophos or phosphinothricin; MAS: mannopine synthase promoter; 35S: cauliflower mosaic virus 35S promoter; *GmIFS*: *Glycine max* L. *isoflavone synthase* gene; ECFP: enhanced cyan fluorescent protein; HA: human influenza hemagglutinin epitope tag; OCS terminator: octopine synthase terminator; pVS1 StaA: stability protein from *Pseudomonas* plasmid pVS1; pVS1 RepA: replication protein from *Pseudomonas* plasmid pVS1; pVS1 OriV: origin of replication for the *Pseudomonas* plasmid; bom: basis of mobility region from pBR322; Ori: origin of replication; and KanR: confers resistance to kanamycin. (**B**) LB and RB: The right and left borders of the T-DNA region in *Agrobacterium*; 35S: cauliflower mosaic virus (CaMV) 35S promoter; P19: the RNA silencing suppressor from tomato bushy stunt virus (TBSV); CPMV RNA-2 5′UTR: 5′ untranslated region from RNA-2 polyprotein of Cowpea mosaic virus (CPMV); CPMV RNA-2 3′UTR: 3′ untranslated region from RNA-2 polyprotein of Cowpea mosaic virus (CPMV); *GmIFS*: *Isoflavone synthase* gene from *Glycine max L*.; OriV: replication origin; ColE1: pBR322 replication origin; TrfA: Replication essential locus; NPT: neomycin phosphotransferase gene conferring resistance to kanamycin; and Nos: nopaline synthase terminator sequence.

The *IFS* gene procured from Donald Danforth Plant Science Center was used as template to clone the gene in plant expression vector pEarleyGate 102 HA (Figure 3A) and pEAQ-HT-DEST 3 (Figure 3B) by gateway cloning. Both the vectors were used for the transient expression of *IFS* gene. The *IFS* gene was cloned into entry vector pDONR/Zeo and further moved into the destination vector pEarleyGate 102 HA and pEAQ-HT-DEST 3. The generated clones were confirmed by polymerase chain reaction (PCR) and restriction digestion.

### 2.2. Transformation of Agrobacterium tumefaciens with GmIFS and Plant Transformation

Plant expression vector pEarleyGate 102 HA and pEAQ-HT-DEST 3 harboring *IFS* gene was mobilized into *A. tumefaciens* AGL1 by electroporation. Electroporation uses short high-voltage pulses which help to beat the capacitance of the cell membrane inducing reversible breakdown of the membrane. This transient, permeabilized state of the membrane allows the passage way of the DNA into the cell [48]. After electroporation, the transformed *A. tumefaciens* colonies were selected on yeast extract and nutrient broth agar plates supplemented with 75 mg L^−1^ kanamycin and 60 mg L^−1^ rifampicin, and the colonies were confirmed by PCR using gene specific primers. Positive clone was further subcultured and used for the agroinfiltration.

**Figure 4:**
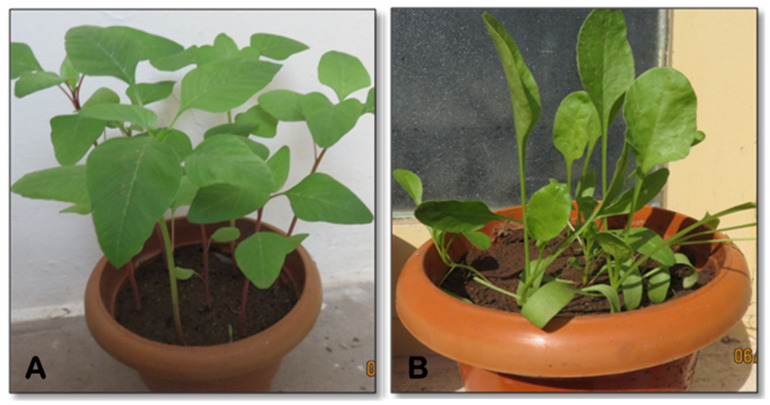
Green leafy vegetables used in the present study. (**A**) *A. tricolor* var. *tristis*; (**B**) *S. oleracea*.

**Figure 5:**
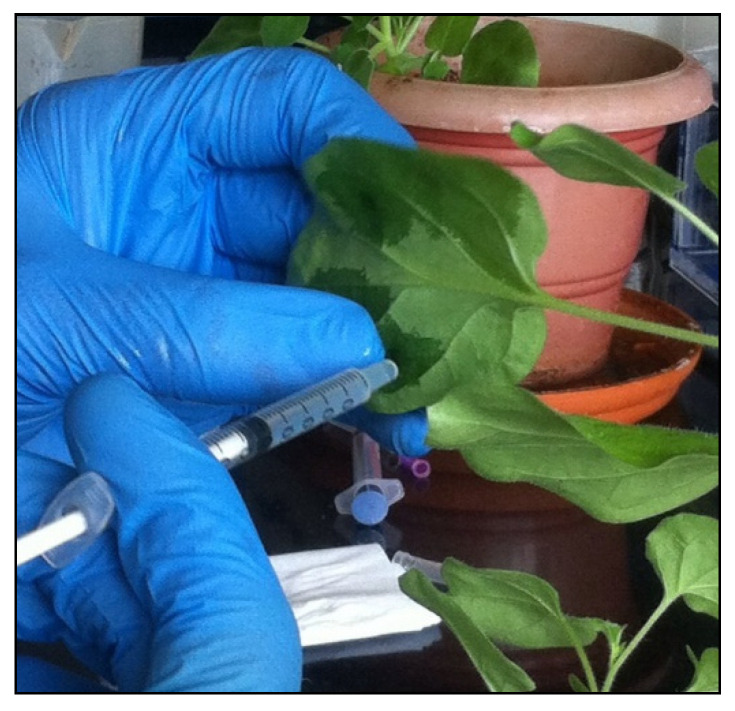
Syringe infiltration; plant leaves were infiltrated using syringe without needle on the abaxial leaf surfaces.

**Figure 6:**
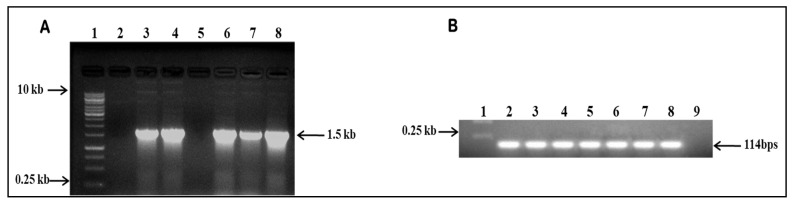
Semiquantitative reverse-transcriptase PCR of *GmIFS* in infiltrated samples. (**A**): Lane 1: DNA Marker; lane 2: *A. tricolor* var. *tristis* control (wild type); lane 3: *A. tricolor* var. *tristis* infiltrated with pEAQ-HT-DEST 3:*IFS*; lane 4: *A. tricolor* var. *tristis* infiltrated with pEarleyGate 102 HA:*IFS*; lane 5: *S. oleracea* control (wild type); lane 6: *S. oleracea* infiltrated with pEAQ-HT-DEST 3:*IFS*; lane 7: *S. oleracea* infiltrated with pEarleyGate HA 102:*IFS*; lane 8: positive control (pEAQ-HT-DEST 3:*IFS* plasmid). (**B**): β-Actin internal control.

We set out to investigate the effect of *IFS* expression in *A. tricolor* var. *tristis* and *S. oleracea* by transient expression using agroinfiltration. Leaves of four-week-old *A. tricolor* var. *tristis* and *S. oleracea* (Figure 4A,B) were infiltrated with *A. tumefaciens* suspensions containing either one of the vectors pEarleyGate 102 HA or pEAQ-HT-DEST 3 harboring *IFS* gene (Figure 5). The infiltrated leaves were harvested at day 4 after infiltration. Total RNA was isolated, and cDNA was synthesized, which was used as the template for the semiquantitative reverse-transcriptase PCR. The expected band size of 1500 bp confirmed the expression of *IFS* gene in the infiltrated leaves of *A. tricolor* var. *tristis* and *S. oleracea* (Figure 6A). The results showed that transcription of the *IFS* gene was similar in the leaves infiltrated either with pEAQ-HT-DEST 3:*IFS* or pEarleyGate 102 HA:*IFS*. β-Actin (~114 bp) was used as an internal control (Figure 6B).

### 2.3. HPLC Detection of Naringenin and Genistein in Infiltrated Samples

**Figure 7:**
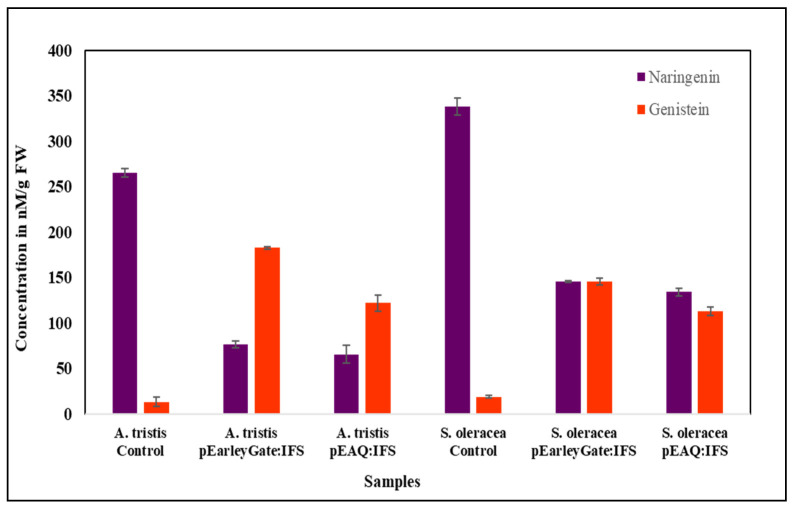
Quantification of naringenin and genistein in control and agroinfiltrated leaf samples of *A. tricolor* var. *tristis* and *S. oleracea*. The HPLC analysis showed the presence of naringenin in control (wild type), and genistein accumulation was observed in agroinfiltrated leaves with pEarleyGate 102 HA:*IFS* and pEAQ-HT-DEST 3:*IFS* in both the green leafy vegetables.

HPLC was performed to determine the presence of naringenin in wild-type *A. tricolor* var. *tristis* and *S. oleracea* followed by genistein detection in the infiltrated leaves. The results showed that the naringenin peaks were observed in both the plant species corresponding to that of naringenin standard. Wild-type *A. tricolor* var. *tristis* and *S. oleracea* showed 265.5 and 338.5 nM/g FW (nanomolar/gram fresh weight) of naringenin in the plant extracts. In the control samples, *S. oleracea* showed higher levels of naringenin content when compared to *A. tricolor* var. *tristis*. (Figure 7). Consequently, the naringenin content was also determined in the agroinfiltrated *A. tricolor* var. *tristis* and *S. oleracea* with pEarleyGate 102 HA and pEAQ-HT-DEST 3 vectors harboring *IFS*. As shown in Figure 7, the leaves of *A. tricolor* var. *tristis* and *S. oleracea* infiltrated with pEarleyGate 102 HA and pEAQ-HT-DEST 3 showed considerably less concentration of naringenin varying in the range of 65.5–134 nM/g FW compared to control.

**Figure 8:**
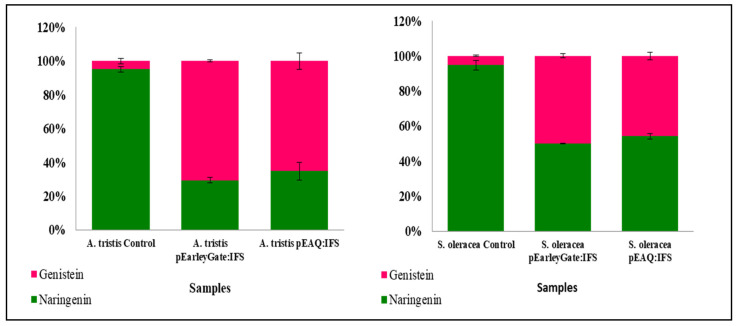
Comparison of genistein accumulation in agroinfiltrated leaves of *A. tricolor* var. *tristis* and *S. oleracea* with pEarleyGate 102 HA:*IFS* and pEAQ-HT-DEST 3:*IFS* with wild type (control).

Quantification analysis showed that the leaves infiltrated with *GmIFS* accumulated comparable amounts of genistein. The peaks in the infiltrated leaves were identified with that of the standard peak of genistein. The wild-type control plants showed a negligible amount of genistein in comparison with the infiltrated leaves. The transiently transformed lines of *A. tricolor* var. *tristis* and *S. oleracea* also showed significant amounts of genistein accumulation with varying concentrations from 113 to 182.6 nM/g FW (Figure 7). It was observed that almost 65% of the concentration of naringenin was utilized as substrate for genistein production in *A. tricolor* var. *tristis* and about 50% in case of *S. oleracea* (Figure 8). The leaves of *A. tricolor* var. *tristis* and *S. oleracea* infiltrated with pEarleyGate 102 HA, and pEAQ-HT-DEST 3 harboring *IFS* showed significant genistein biosynthesis (Figure 8).

## 3. Discussion

The phenylpropanoid pathway is one of the most studied metabolic frameworks that fulfills several physiological functions necessary for plant growth and development. This pathway involves an array of enzymes that are responsible for the production of different metabolites. *IFS* is one among them, as it catalyzes the synthesis of isoflavonoids genistein, daidzein from flavonoids naringenin and liquiritigenin, respectively, by an aryl ring migration [18,49,50]. As these isoflavonoids are reported to benefit plethora of beneficial activities in humans [3,8,11,14,18,50,51], the present research emphasized on engineering the metabolic pathway by transient expression of *GmIFS* in non-leguminous plants *A. tricolor* var. *tristis* and *S. oleracea* for the production of genistein which is not normally produced by this species.

Metabolic engineering holds a promising hope for the viable systematization of production of biologically active compounds that are necessary for human health and generation of improved crop varieties involving the redirection of enzymatic reaction(s) in endogenous pathways to synthesize novel products or enrich the existing ones [52,53,54,55]. Two approaches employed for the functional gene studies in plants are stable and transient expression. The development of stable transgenic lines by the stable integration of genome of plants is a time-consuming and laborious process. By contrast, the transient expression of heterologous genes in plants has considerable advantages over the stable expression, which is quick and inexpensive for the production of recombinant biopharmaceuticals and valuable metabolites [26,46]. Further, this approach could be utilized to study the activity of endogenous and exogenous enzymes in metabolic engineering, which provides insight to understand the mechanisms underlying in the complex gene regulatory and metabolic pathways. The transient gene expression is a rapid, flexible and cost-effective approach to study the expression of heterologous genes without the integration of desired gene(s) in the host genome [56,57]. The transient expression can be performed either by syringe infiltration or vacuum infiltration [58,59,60]. The high level of foreign gene expression can be obtained by co-expression of a viral-encoded suppressor of gene silencing which prevents the post-transcriptional gene silencing in the infiltrated tissues [25,30].

Here, we have introduced the *GmIFS* gene in pEarleyGate HA 102 and pEAQ-HT-DEST 3 by gateway cloning [21,61] and mobilized the vectors into *A. tumefaciens* for agroinfiltration in *A. tricolor* var. *tristis* and *S. oleracea* leaves. Further, the gene expression was confirmed by RT-PCR. The expected amplicon size of ~1.5 kb corresponding to *IFS* gene was observed which confirmed the heterologous expression of *IFS* in the infiltrated leaf samples of *A. tricolor* var. *tristis* and *S. oleracea*. The results suggested that the isoflavonoid synthesis is catalyzed by the presence of *IFS* using the flavonoid intermediates [18,19,21]. The earlier reports showed that the stable expression of *IFS* gene in non-legume plants such as rice, tomato, *Arabidopsis*, *Petunia* and onion can produce genistein in transgenic plants [19,21,62,63,64]. The HPLC results revealed that levels of naringenin in the leaf samples of infiltrated *A. tricolor* var. *tristis* and *S. oleracea* decreased significantly compared to that of control, indicating that this compound was utilized by the transformed *IFS* gene for genistein production. However, the bioactivity of genistein produced transiently in both the plants was not examined. The future work will focus on the elucidation of genistein activity and its production in crop plants. The present study demonstrated that both the plants could be used as hosts for the analysis and pathway engineering of metabolic enzymes. It also depicts that the transient system can be employed to study the function of new vector and validate the expression of both endogenous and exogenous enzymes in metabolic pathways in short time.

## 4. Materials and Methods

### 4.1. Plant Materials

The seeds of *A. tricolor* var. *tristis* and *S. oleracea* were procured from the seed center, Tamil Nadu Agricultural University, Coimbatore, India. These seeds were grown in soil under greenhouse conditions. The plantlets were allowed to grow for 4–5 weeks.

### 4.2. Construction of Plant Expression Vector

*IFS* cDNA (Genbank accession number: AF195798) was kindly provided by Dr. Oliver Yu and Dr. Brian Mc Gonigle, Donald Danforth Plant Science Center, USA. The *IFS* gene was cloned into plant expression vector pEarleyGate 102 HA and pEAQ-HT-DEST 3 by gateway cloning technology (Invitrogen, Carlsbad, CA, USA) as per manufacturer’s protocol. The *IFS* gene was amplified by PCR, and purified PCR products were cloned into entry vector pDONR/Zeo and moved to destination vectors pEarleyGate 102 HA and pEAQ-HT-DEST 3, as described previously [21]. The transformed colonies in the destination vector were confirmed by gene-specific PCR and restriction digestion. Subsequently, the plant expression vectors were transformed to *A. tumefaciens* strain AGL1 by electroporation.

### 4.3. Agroinfiltration

*A. tumefaciens* strain AGL1 either containing pEarleyGate 102 HA or pEAQ-HT-DEST 3 plasmid harboring *IFS* gene under the control of CaMV 35S promoter were used for transient expression. The *A. tumefaciens* cultures were inoculated in 5 mL of yeast extract, and nutrient broth medium was supplemented with 60 mg L^−1^ rifampicin and 75 mg L^−1^ kanamycin and incubated at 28 °C for 2 days. The grown culture was centrifuged at 5000× *g* rpm for 15 min at 4 °C. The supernatants were discarded, and pellets were resuspended in Murashige and Skoog liquid medium. Acetosyringone (150 µM) was added to this suspension and incubated at room temperature for 2 h. *Agrobacterium* suspension was infiltrated into the leaves of *A. tricolor* var. *tristis* and *S. oleracea* leaf surfaces using the syringe infiltration technique [65]. The healthy leaves were infiltrated using 1 mL syringe without the needle on the abaxial leaf surfaces with *Agrobacterium* suspension to spread throughout the leaf lamina and air spaces. The infiltrated plants were maintained at 24 °C with a 16 h light cycle for 4 days.

### 4.4. RNA Isolation and cDNA Synthesis

All the materials used for the RNA isolation were treated with diethyl pyrocarbonate (Sigma-Aldrich, Saint Louis, MO, USA). The total RNA was isolated from the infiltrated leaves of *A. tricolor* var. *tristis* and *S. oleracea* after 4 days post infiltration (dpi). The infiltrated leaves and wild-type leaves were harvested and immediately frozen in liquid nitrogen. The total RNA was isolated from the leaves using total RNA isolation kit (Macherey-Nagel, Düren, Germany). The cDNA was synthesized from the total RNA with random hexamer primer using the M-MuLV reverse transcriptase (M-MuLV RTase) according to the manufacturer’s instructions (Fermentas, Waltham, MA, USA). The reaction components used for the cDNA synthesis consisted of 1 μg RNA, 0.5 μL of random hexamer, nuclease-free water, and the reaction mixture was incubated at 65 °C for 2 min for denaturation. Immediately, it was kept in ice for 2 min, and the enzyme mixture containing 1U of M-MuLV RTase, buffer, dNTPs were added and kept at 37 °C for one hour and then inactivated at 95 °C for 5 min. The synthesized cDNA was used as template for PCR using the *IFS* gene specific primers. A reaction mixture containing the PCR master mix, primers and DNA template was prepared as mentioned in Table 1. β-*Actin* was used as internal control for the semiquantitative expression analyses.

### 4.5. HPLC Detection

The naringenin standard was obtained from Sigma Aldrich for HPLC analysis. After 4 days post infiltration, infiltrated leaf samples of *A. tricolor* var. *tristis* and *S. oleracea* were collected. Wild-type leaves from both the plants were collected and used as control. One gram of fresh leaves was homogenized in HPLC grade methanol. The homogenized mixture was incubated in a concentrator plus vacuum evaporator (Eppendorf, Germany) at 55 °C for 6–8 h. The dried samples were again extracted in HPLC methanol and filtered through 0.45 μm filter. Waters HPLC system equipped with a model 515 HPLC pump, Model 2998 photodiode-array detector and auto sampler was used for analysis. Separations were performed on a C_18_ column with acetonitrile-water as mobile phase, and the reaction conditions were followed as described previously [21]. Naringenin in the samples was detected at 280 nm and identified by comparison of their retention times with those of standard.

The genistein standard was obtained from Sigma Aldrich for HPLC detection. Wild-type leaves from *A. tricolor* var. *tristis* and *S. oleracea* were used as controls. After 4 dpi, leaf samples of *A. tricolor* var. *tristis* and *S. oleracea* leaves were collected, homogenized in HPLC grade methanol and evaporated in concentrator plus vacuum evaporator (Eppendorf, Germany) at 55 °C for 6–8 h. The obtained dried samples were reconstituted in methanol, and 0.45 μL filtration was performed for HPLC analysis. The mobile phase was 80% methanol programmed at a flow rate of 1.0 mL/minute [51]. The injection volume was 20 μL, and the column was maintained at ambient temperature (24–26 °C). Genistein in the samples was detected at 260 nm and identified by comparison of their retention times with those of standard.

### 4.6. Statistical Analysis

Two biological replicates were used for HPLC quantification. The results were expressed as mean ± standard deviation.

## 5. Conclusions

The present study demonstrated that the transient expression of *GmIFS* gene in *A. tricolor* var. *tristis* and *S. oleracea* can produce the isoflavone genistein from the intermediate naringenin. The transient approach has been utilized to introduce the isoflavone pathway in these green leafy vegetables. To the best of our knowledge, this is the first report on transient expression of *GmIFS* in the *A. tricolor* var. *tristis* and *S. oleracea* for genistein production. The phenyl propanoid pathway being a complex metabolic pathway can be further explored in detail by the transient approach with infiltration or co-infiltration of multiple genes for complete understanding of the role of other enzymes and substrates involved in biosynthesis of isoflavonoids and other secondary metabolites. This proof-of-concept study deciphers light on the production of health-beneficial isoflavones, and other pharmaceutically and nutraceutically relevant compounds by metabolic engineering, which can be employed to produce biofortified crops with better nutritional value for human health.

## Figures and Tables

**Table 1:** PCR Components and reaction conditions used in the present study.

Reaction Mix		Program	
Components	Volume (µL)	1. Initial Denaturation	94 °C for 5 min
Emerald AmpR PCR master mix	10	2. Denaturation	94 °C for 30 s
Forward primer (10 pM)	1	3. Annealing	56 °C for 45 s
Reverse primer (10 pM)	1	4. Extension	72 °C for 45 s
Nuclease-free water	6	Step 2–4 repeated 25 cycles	
DNA template	2	Final Extension	72 °C for 10 min
Total	20	Final Hold	4 °C

## Data Availability

The data presented in this study are available on request from the corresponding author.

## Abbreviations

CaMV	Cauliflower mosaic virus
cDNA	Complementary deoxyribonucleic acid
DNA	Deoxyribonucleic acid
dNTP’s	Deoxynucleotide triphosphates
°C	Degrees Celsius
FW	Fresh weight
HPLC	High-performance liquid chromatography
mg L^−1^	Milligram per liter
mL	Milliliter
nm	Nanometer
RNA	Ribonucleic acid
rpm	Revolutions per minute
µL	Microliter
µM	Micromolar
µ	Micron
U	Unit
